# (4-Chloro­benzoyl)(2-eth­oxy-7-methoxy­naphthalen-1-yl)methanone

**DOI:** 10.1107/S1600536809004796

**Published:** 2009-02-18

**Authors:** Ryosuke Mitsui, Keiichi Noguchi, Noriyuki Yonezawa

**Affiliations:** a Department of Organic and Polymer Materials Chemistry, Tokyo University of Agriculture and Technology, 2-24-16 Naka-machi, Koganei, Tokyo 184-8588, Japan; b Instrumentation Analysis Center, Tokyo University of Agriculture and Technology, 2-24-16 Naka-machi, Koganei, Tokyo 184-8588, Japan

## Abstract

In the title compound, C_20_H_17_ClO_3_, the naphthalene and benzene rings form an inter­planar angle of 83.30 (8)°. The conformation around the central C=O group is such that the C=O bond vector forms a larger angle to the plane of the naphthalene ring than to the plane of the benzene ring, *viz*. 55.8 (2)° *versus* 15.8 (2)°. The 4-chloro­phenyl groups form a centrosymmetric π–π inter­action, with a centroid–centroid distance of 3.829 (1) Å and a lateral offset of 1.758 Å. An inter­molecular C—H⋯O inter­action is formed between the 4-chloro­phenyl group and the O atom of a neighbouring meth­oxy group, and two very weak C—H⋯π contacts are present.

## Related literature

For structures of closely related compounds, see: Mitsui, Nakaema, Noguchi, Okamoto & Yonezawa (2008 ▶); Mitsui, Nakaema, Noguchi & Yonezawa (2008 ▶).
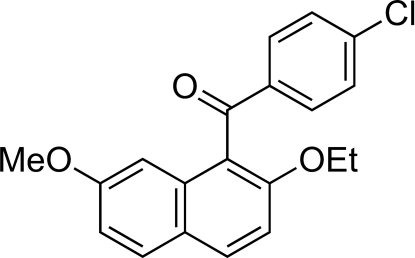


## Experimental

### 

#### Crystal data

C_20_H_17_ClO_3_
*M* *_r_* = 340.79
Monoclinic, 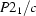

*a* = 7.26434 (13) Å
*b* = 20.8849 (4) Å
*c* = 12.2094 (2) Å
β = 113.201 (1)°
*V* = 1702.55 (5) Å^3^
*Z* = 4
Cu *K*α radiation
μ = 2.11 mm^−1^
*T* = 193 K
0.40 × 0.30 × 0.20 mm

#### Data collection

Rigaku R-AXIS RAPID diffractometer
Absorption correction: numerical (*NUMABS*; Higashi, 1999 ▶) *T*_min_ = 0.542, *T*_max_ = 0.656
30947 measured reflections
3104 independent reflections
2544 reflections with *I* > 2σ(*I*)
*R*_int_ = 0.023

#### Refinement

*R*[*F* ^2^ > 2σ(*F* ^2^)] = 0.041
*wR*(*F* ^2^) = 0.120
*S* = 1.10
3104 reflections
218 parameters
23 restraints
H-atom parameters constrained
Δρ_max_ = 0.27 e Å^−3^
Δρ_min_ = −0.28 e Å^−3^

### 

Data collection: *PROCESS-AUTO* (Rigaku, 1998 ▶); cell refinement: *PROCESS-AUTO*; data reduction: *CrystalStructure* (Rigaku/MSC, 2004 ▶); program(s) used to solve structure: *SIR2004* (Burla *et al.*, 2005 ▶); program(s) used to refine structure: *SHELXL97* (Sheldrick, 2008 ▶); molecular graphics: *ORTEPIII* (Burnett & Johnson, 1996 ▶); software used to prepare material for publication: *SHELXL97*.

## Supplementary Material

Crystal structure: contains datablocks global, I. DOI: 10.1107/S1600536809004796/bi2344sup1.cif

Structure factors: contains datablocks I. DOI: 10.1107/S1600536809004796/bi2344Isup2.hkl

Additional supplementary materials:  crystallographic information; 3D view; checkCIF report

## Figures and Tables

**Table 1:** Hydrogen-bond geometry (Å, °)

*D*—H⋯*A*	*D*—H	H⋯*A*	*D*⋯*A*	*D*—H⋯*A*
C20—H20*B*⋯*Cg*1^i^	0.98	3.02	3.821 (3)	140
C20—H20*C*⋯*Cg*1^ii^	0.98	3.01	3.477 (3)	110
C13—H13⋯O3^iii^	0.95	2.44	3.213 (2)	138

Symmetry codes: (i) 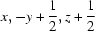; (ii) 
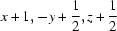
; (iii) 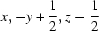. *Cg*1 is the centroid of the C1–C5/C10 ring.

**Fig. 1.**
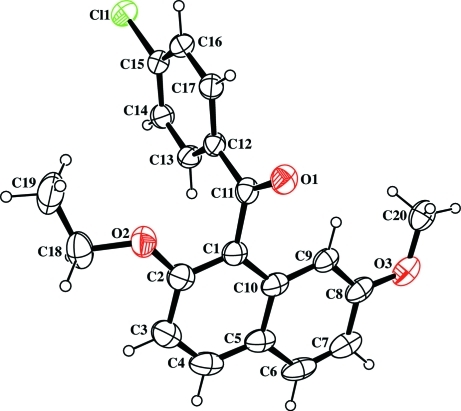
Molecular structure with displacement ellipsoids at 30% probability for non-H atoms.

**Fig. 2.**
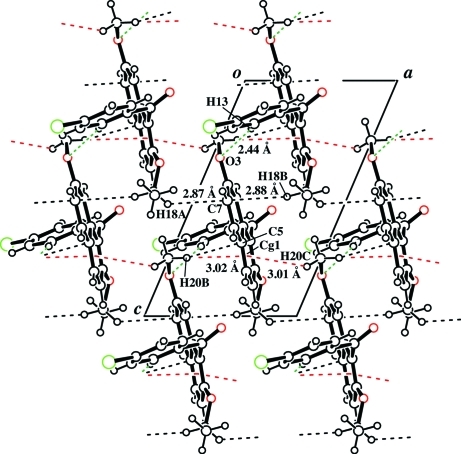
Packing diagram viewed down the b axis. Van der Waals, C—H···π and C—H···O interactions are shown as black, red and green dashed lines, respectively.

**Fig. 3.**
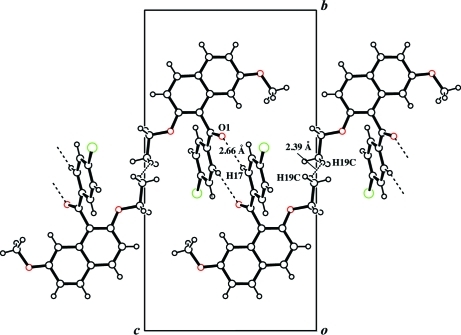
Partial packing diagram viewed down the a axis. Van der Waals interactions are shown as dashed lines.

**Fig. 4.**
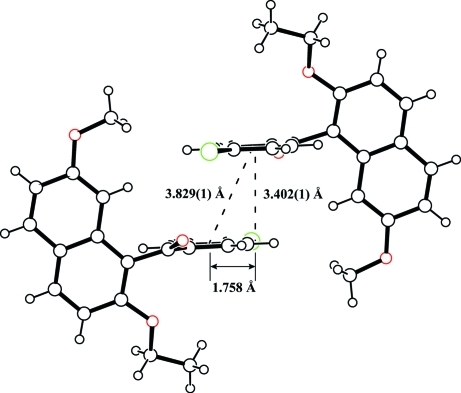
Side-on view of the π–π interaction.

## supplementary crystallographic information

### Comment

Recently, we have reported the crystal structures of 1-(4-chlorobenzoyl)-2,7-dimethoxynaphthalene and (4-chlorophenyl)(2-hydroxy-7-methoxynaphthalen-1-yl)methanone (Mitsui, Nakaema, Noguchi, Okamoto & Yonezawa, 2008; Mitsui, Nakaema, Noguchi & Yonezawa, 2008). As a part of our ongoing studies on the synthesis and crystal structure analyses of aroylated naphthalene derivatives, this paper reports the crystal structure of the title compound, prepared by ethylation of (4-chlorophenyl)(2-hydroxy-7-methoxynaphthalen-1-yl)methanone with ethyl iodide.

In the molecule (Fig. 1), the interplanar angle between the benzene ring [C12–C17] and the naphthalene ring [C1–C10] is 83.30 (8)°. The C=O bond vector lies close to the mean plane of the benzene ring (angle 15.8 (2)°), but forms an angle of 55.77 (15)° to the plane of the naphthalene ring. The conformation of these groups is similar to 1-(4-chlorobenzoyl)-2,7-dimethoxynaphthalene. On the other hand, the methoxy group is arranged toward the aroyl group [C20—O3—C8—C7 torsion angle = 177.7 (2)°] while that of the aforementioned related compound is arranged toward the naphthalene ring [-7.1 (3)°]. In both compounds, the C—O bond vector of the methoxy group lies approximately in the plane of the naphthalene ring [angle 4.2 (1)° in the title compound, 5.05 (9)° in the related compound].

In the crystal structure, the naphthalene rings interact with ethyl groups [C7···H18A = 2.87 Å, C7···H18B = 2.88 Å] and methyl groups [C5···H20B = 2.75 Å] of the adjacent molecule along the *a* axis (Fig. 2). The neighboring inversion-related ethyl groups interact with each other [H19C···H19C = 2.39 Å] along the *c* axis. The C=O groups interact with benzene rings [O1···H17 = 2.66 Å] along the *b* axis (Fig. 3). Adjacent 4-chlorophenyl groups related by crystallographic inversion centers are exactly antiparallel and the perpendicular distance between the mean planes of these groups is 3.402 (1) Å (Fig. 4). The centroid–centroid distance between the two antiparallel phenyl rings is 3.829 (1) Å and the lateral offset is 1.758 Å, indicating the presence of a π–π interaction. Moreover, molecules are linked by C—H···π interactions. The methyl group acts as a hydrogen-bond donor and the π system of the naphthalene ring [C1/C2/C3/C4/C5/C10 ring (with centroid *Cg1*)] of an adjacent molecule acts as an acceptor, *viz.* C20—H20B···π, C20—H20C···π (Fig. 2 and Table 1). Intermolecular C—H···O hydrogen bonds between the methoxy O and an H atom of the 4-chlorophenyl group of the adjacent molecule are also found along the *c* axis (C13—H13···O3^i^; Fig. 2 and Table 1).

### Experimental

(4-Chlorophenyl)(2-hydroxy-7-methoxynaphthalen-1-yl)methanone (0.13 g, 0.40 mmol) was dissolved in acetone (1.0 ml) and aqueous 0.8M NaOH (1.0 ml). EtI (0.31 g, 2.0 mmol) was added and the reaction mixture was heated at reflux for 6 h. Upon cooling to ambient temperature, the mixture was poured into H_2_O (5 ml) and CHCl_3_ (5 ml), and the aqueous layer was extracted with CHCl_3_ (3 × 5 ml). The combined organic layers were washed with brine (3 × 20 ml), and dried over MgSO_4_ overnight. The solvent was removed *in vacuo* and the crude material was purified by recrystallization from hexanes to give the title compound as colorless blocks (m.p. 365.5–366.0 K, yield 95 mg, 70%).

Spectroscopic Data: ^1^H NMR (300 MHz, CDCl_3_) δ 7.83 (d, 1H), 7.77 (d, 2H), 7.70 (d, 1H), 7.38 (d, 2H), 7.11 (d, 1H), 7.02 (dd, 1H), 6.86 (d, 1H), 4.05 (q, 2H), 3.74 (s, 3H), 1.10 (s, 3H); ^13^C NMR (75 MHz, CDCl_3_) δ 196.8, 159.1, 154.8, 139.4, 137.1, 133.2, 131.3, 130.7, 129.7, 128.7, 124.5, 121.7, 117.2, 111.5, 102.3, 65.0, 55.2, 14.6; IR (KBr): 1671, 1624, 1582, 1511, 1464, 1249, 1227, 1046; HRMS (*m*/*z*): [*M* + H]^+^ calcd for C_20_H_18_ClO_3_, 341.0945; found, 341.0903.

### Refinement

Rigid bond restraints were applied to the *U*_ij_ values of the naphthalene ring (C4—C7) (5 restraints with the DELU command in *SHELXL97*). Further restraints were used to generate similar *U*_ij_ values for the atoms of naphthalene ring (18 restraints with the SIMU command in *SHELXL97*). All H atoms were visible in difference maps but were subsequently placed in calculated positions and refined as riding, with C—H = 0.95 (aromatic), 0.98 (methyl) and 0.99 (methylene) Å, and with *U*_iso_(H) = 1.2*U*_eq_(C).

### Crystal data

**Table:**

C_20_H_17_ClO_3_	*F*(000) = 712
*M**_r_* = 340.79	*D*_x_ = 1.330 Mg m^−^^3^
Monoclinic, *P*2_1_/*c*	Melting point = 365.5–366.0 K
Hall symbol: -P 2ybc	Cu *K*α radiation, λ = 1.54187 Å
*a* = 7.26434 (13) Å	Cell parameters from 26200 reflections
*b* = 20.8849 (4) Å	θ = 3.9–68.1°
*c* = 12.2094 (2) Å	µ = 2.11 mm^−^^1^
β = 113.201 (1)°	*T* = 193 K
*V* = 1702.55 (5) Å^3^	Block, colorless
*Z* = 4	0.40 × 0.30 × 0.20 mm

### Data collection

**Table:**

Rigaku R-AXIS RAPID diffractometer	3104 independent reflections
Radiation source: rotating anode	2544 reflections with *I* > 2σ(*I*)
graphite	*R*_int_ = 0.023
Detector resolution: 10.00 pixels mm^-1^	θ_max_ = 68.1°, θ_min_ = 4.2°
ω scans	*h* = −8→8
Absorption correction: numerical (*NUMABS*; Higashi, 1999)	*k* = −25→25
*T*_min_ = 0.542, *T*_max_ = 0.656	*l* = −14→14
30947 measured reflections	

### Refinement

**Table:**

Refinement on *F*^2^	Secondary atom site location: difference Fourier map
Least-squares matrix: full	Hydrogen site location: inferred from neighbouring sites
*R*[*F*^2^ > 2σ(*F*^2^)] = 0.041	H-atom parameters constrained
*wR*(*F*^2^) = 0.120	*w* = 1/[σ^2^(*F*_o_^2^) + (0.0592*P*)^2^ + 0.3688*P*] where *P* = (*F*_o_^2^ + 2*F*_c_^2^)/3
*S* = 1.10	(Δ/σ)_max_ = 0.001
3104 reflections	Δρ_max_ = 0.27 e Å^−^^3^
218 parameters	Δρ_min_ = −0.28 e Å^−^^3^
23 restraints	Extinction correction: *SHELXL97* (Sheldrick, 2008), Fc^*^=kFc[1+0.001xFc^2^λ^3^/sin(2θ)]^-1/4^
Primary atom site location: structure-invariant direct methods	Extinction coefficient: 0.0020 (3)

### Special details

**Table:**

Geometry. All e.s.d.'s (except the e.s.d. in the dihedral angle between two l.s. planes) are estimated using the full covariance matrix. The cell e.s.d.'s are taken into account individually in the estimation of e.s.d.'s in distances, angles and torsion angles; correlations between e.s.d.'s in cell parameters are only used when they are defined by crystal symmetry. An approximate (isotropic) treatment of cell e.s.d.'s is used for estimating e.s.d.'s involving l.s. planes.
Refinement. Refinement of *F*^2^ against ALL reflections. The weighted *R*-factor *wR* and goodness of fit *S* are based on *F*^2^, conventional *R*-factors *R* are based on *F*, with *F* set to zero for negative *F*^2^. The threshold expression of *F*^2^ > σ(*F*^2^) is used only for calculating *R*-factors(gt) *etc*. and is not relevant to the choice of reflections for refinement. *R*-factors based on *F*^2^ are statistically about twice as large as those based on *F*, and *R*- factors based on ALL data will be even larger.

### Fractional atomic coordinates and isotropic or equivalent isotropic displacement parameters (Å^2^)

**Table:**

	*x*	*y*	*z*	*U*_iso_*/*U*_eq_	
Cl1	1.10278 (9)	0.57118 (2)	0.30495 (5)	0.0781 (2)	
O1	0.4618 (2)	0.39338 (6)	0.45026 (12)	0.0659 (4)	
O2	0.3297 (2)	0.37751 (7)	0.15066 (12)	0.0756 (4)	
O3	0.9489 (2)	0.19765 (7)	0.67286 (15)	0.0765 (4)	
C1	0.5171 (3)	0.31905 (9)	0.31945 (17)	0.0566 (4)	
C2	0.4068 (3)	0.31950 (10)	0.19824 (18)	0.0656 (5)	
C3	0.3762 (3)	0.26240 (12)	0.1314 (2)	0.0767 (6)	
H3	0.3019	0.2628	0.0476	0.092*	
C4	0.4553 (3)	0.20662 (11)	0.1892 (2)	0.0784 (7)	
H4	0.4326	0.1681	0.1443	0.094*	
C5	0.5692 (3)	0.20400 (9)	0.3128 (2)	0.0671 (5)	
C6	0.6600 (4)	0.14645 (10)	0.3747 (3)	0.0794 (7)	
H6	0.6349	0.1072	0.3321	0.095*	
C7	0.7796 (4)	0.14653 (10)	0.4912 (3)	0.0785 (6)	
H7	0.8367	0.1075	0.5296	0.094*	
C8	0.8208 (3)	0.20409 (9)	0.5568 (2)	0.0665 (5)	
C9	0.7332 (3)	0.26027 (8)	0.50295 (18)	0.0572 (4)	
H9	0.7589	0.2987	0.5482	0.069*	
C10	0.6044 (3)	0.26155 (8)	0.38026 (18)	0.0579 (5)	
C11	0.5461 (3)	0.38232 (8)	0.38384 (15)	0.0536 (4)	
C12	0.6830 (3)	0.42954 (8)	0.36347 (15)	0.0505 (4)	
C13	0.8197 (3)	0.40999 (8)	0.31679 (16)	0.0547 (4)	
H13	0.8235	0.3663	0.2963	0.066*	
C14	0.9509 (3)	0.45307 (9)	0.29947 (16)	0.0587 (4)	
H14	1.0453	0.4393	0.2684	0.070*	
C15	0.9411 (3)	0.51637 (8)	0.32839 (15)	0.0569 (4)	
C16	0.8073 (3)	0.53732 (9)	0.37524 (16)	0.0599 (5)	
H16	0.8032	0.5812	0.3945	0.072*	
C17	0.6795 (3)	0.49384 (8)	0.39383 (16)	0.0569 (4)	
H17	0.5887	0.5077	0.4275	0.068*	
C18	0.2883 (4)	0.38987 (15)	0.0278 (2)	0.0872 (7)	
H18A	0.1616	0.3689	−0.0242	0.105*	
H18B	0.3980	0.3735	0.0067	0.105*	
C19	0.2714 (5)	0.46071 (17)	0.0136 (3)	0.1199 (11)	
H19A	0.2428	0.4720	−0.0695	0.144*	
H19B	0.3976	0.4806	0.0658	0.144*	
H19C	0.1626	0.4761	0.0350	0.144*	
C20	1.0050 (3)	0.25362 (11)	0.7447 (2)	0.0754 (6)	
H20A	1.0970	0.2420	0.8256	0.090*	
H20B	0.8853	0.2738	0.7476	0.090*	
H20C	1.0715	0.2837	0.7105	0.090*	

### Atomic displacement parameters (Å^2^)

**Table:**

	*U*^11^	*U*^22^	*U*^33^	*U*^12^	*U*^13^	*U*^23^
Cl1	0.0934 (4)	0.0670 (3)	0.0780 (4)	−0.0205 (3)	0.0382 (3)	0.0047 (2)
O1	0.0784 (9)	0.0658 (8)	0.0721 (8)	0.0068 (7)	0.0496 (7)	0.0002 (6)
O2	0.0841 (10)	0.0855 (10)	0.0595 (8)	−0.0073 (8)	0.0306 (7)	0.0064 (7)
O3	0.0712 (9)	0.0656 (9)	0.1016 (11)	0.0101 (7)	0.0435 (9)	0.0261 (8)
C1	0.0633 (11)	0.0567 (10)	0.0629 (11)	−0.0068 (8)	0.0390 (9)	−0.0048 (8)
C2	0.0665 (12)	0.0731 (13)	0.0699 (12)	−0.0139 (10)	0.0406 (10)	−0.0083 (10)
C3	0.0749 (14)	0.0969 (17)	0.0735 (13)	−0.0280 (12)	0.0457 (11)	−0.0229 (12)
C4	0.0799 (14)	0.0744 (14)	0.1077 (17)	−0.0300 (12)	0.0658 (14)	−0.0359 (13)
C5	0.0698 (12)	0.0585 (11)	0.0970 (15)	−0.0162 (9)	0.0586 (12)	−0.0188 (10)
C6	0.0850 (15)	0.0482 (11)	0.138 (2)	−0.0128 (10)	0.0796 (16)	−0.0187 (12)
C7	0.0798 (15)	0.0507 (11)	0.128 (2)	0.0019 (10)	0.0655 (15)	0.0056 (12)
C8	0.0659 (12)	0.0546 (11)	0.1001 (16)	0.0007 (9)	0.0554 (12)	0.0089 (10)
C9	0.0637 (11)	0.0479 (9)	0.0766 (12)	0.0002 (8)	0.0453 (10)	0.0028 (8)
C10	0.0628 (11)	0.0496 (9)	0.0820 (12)	−0.0056 (8)	0.0507 (10)	−0.0057 (8)
C11	0.0607 (10)	0.0541 (10)	0.0527 (9)	0.0091 (8)	0.0295 (8)	0.0040 (7)
C12	0.0617 (10)	0.0464 (9)	0.0483 (9)	0.0066 (7)	0.0270 (8)	0.0026 (7)
C13	0.0677 (11)	0.0456 (9)	0.0600 (10)	0.0003 (8)	0.0351 (9)	−0.0031 (7)
C14	0.0658 (11)	0.0593 (10)	0.0589 (10)	−0.0008 (8)	0.0332 (9)	−0.0021 (8)
C15	0.0676 (11)	0.0525 (9)	0.0491 (9)	−0.0038 (8)	0.0213 (8)	0.0060 (7)
C16	0.0758 (12)	0.0439 (9)	0.0585 (10)	0.0067 (8)	0.0247 (9)	0.0043 (7)
C17	0.0691 (11)	0.0508 (9)	0.0550 (9)	0.0120 (8)	0.0290 (9)	0.0019 (7)
C18	0.0657 (13)	0.134 (2)	0.0608 (12)	−0.0117 (13)	0.0243 (10)	0.0111 (13)
C19	0.119 (2)	0.142 (3)	0.0847 (18)	−0.014 (2)	0.0247 (16)	0.0474 (18)
C20	0.0702 (13)	0.0782 (14)	0.0890 (15)	0.0106 (11)	0.0434 (12)	0.0189 (12)

### Geometric parameters (Å, °)

**Table:**

Cl1—C15	1.7426 (19)	C9—H9	0.950
O1—C11	1.215 (2)	C11—C12	1.490 (3)
O2—C2	1.365 (3)	C12—C13	1.387 (2)
O2—C18	1.432 (3)	C12—C17	1.396 (2)
O3—C8	1.361 (3)	C13—C14	1.386 (3)
O3—C20	1.421 (3)	C13—H13	0.950
C1—C2	1.378 (3)	C14—C15	1.377 (3)
C1—C10	1.423 (3)	C14—H14	0.950
C1—C11	1.509 (2)	C15—C16	1.379 (3)
C2—C3	1.413 (3)	C16—C17	1.380 (3)
C3—C4	1.366 (4)	C16—H16	0.950
C3—H3	0.950	C17—H17	0.950
C4—C5	1.408 (3)	C18—C19	1.489 (4)
C4—H4	0.950	C18—H18A	0.990
C5—C10	1.422 (3)	C18—H18B	0.990
C5—C6	1.435 (3)	C19—H19A	0.980
C6—C7	1.343 (3)	C19—H19B	0.980
C6—H6	0.950	C19—H19C	0.980
C7—C8	1.410 (3)	C20—H20A	0.980
C7—H7	0.950	C20—H20B	0.980
C8—C9	1.373 (3)	C20—H20C	0.980
C9—C10	1.420 (3)		
			
C2—O2—C18	119.19 (18)	C13—C12—C11	120.47 (15)
C8—O3—C20	118.27 (16)	C17—C12—C11	120.62 (16)
C2—C1—C10	121.15 (17)	C14—C13—C12	121.21 (16)
C2—C1—C11	117.15 (17)	C14—C13—H13	119.4
C10—C1—C11	121.68 (16)	C12—C13—H13	119.4
O2—C2—C1	115.52 (17)	C15—C14—C13	118.40 (17)
O2—C2—C3	124.0 (2)	C15—C14—H14	120.8
C1—C2—C3	120.5 (2)	C13—C14—H14	120.8
C4—C3—C2	118.9 (2)	C14—C15—C16	121.81 (17)
C4—C3—H3	120.6	C14—C15—Cl1	118.81 (15)
C2—C3—H3	120.6	C16—C15—Cl1	119.38 (14)
C3—C4—C5	122.49 (19)	C15—C16—C17	119.29 (17)
C3—C4—H4	118.8	C15—C16—H16	120.4
C5—C4—H4	118.8	C17—C16—H16	120.4
C4—C5—C10	118.9 (2)	C16—C17—C12	120.37 (17)
C4—C5—C6	123.5 (2)	C16—C17—H17	119.8
C10—C5—C6	117.6 (2)	C12—C17—H17	119.8
C7—C6—C5	121.9 (2)	O2—C18—C19	106.0 (2)
C7—C6—H6	119.1	O2—C18—H18A	110.5
C5—C6—H6	119.1	C19—C18—H18A	110.5
C6—C7—C8	120.4 (2)	O2—C18—H18B	110.5
C6—C7—H7	119.8	C19—C18—H18B	110.5
C8—C7—H7	119.8	H18A—C18—H18B	108.7
O3—C8—C9	125.51 (19)	C18—C19—H19A	109.5
O3—C8—C7	114.35 (19)	C18—C19—H19B	109.5
C9—C8—C7	120.1 (2)	H19A—C19—H19B	109.5
C8—C9—C10	120.65 (18)	C18—C19—H19C	109.5
C8—C9—H9	119.7	H19A—C19—H19C	109.5
C10—C9—H9	119.7	H19B—C19—H19C	109.5
C9—C10—C5	119.24 (18)	O3—C20—H20A	109.5
C9—C10—C1	122.64 (16)	O3—C20—H20B	109.5
C5—C10—C1	118.02 (19)	H20A—C20—H20B	109.5
O1—C11—C12	122.05 (16)	O3—C20—H20C	109.5
O1—C11—C1	120.71 (16)	H20A—C20—H20C	109.5
C12—C11—C1	117.24 (14)	H20B—C20—H20C	109.5
C13—C12—C17	118.90 (16)		
			
C18—O2—C2—C1	154.21 (17)	C6—C5—C10—C1	179.21 (16)
C18—O2—C2—C3	−26.8 (3)	C2—C1—C10—C9	174.41 (16)
C10—C1—C2—O2	179.52 (15)	C11—C1—C10—C9	−3.8 (3)
C11—C1—C2—O2	−2.2 (2)	C2—C1—C10—C5	−2.0 (3)
C10—C1—C2—C3	0.5 (3)	C11—C1—C10—C5	179.83 (16)
C11—C1—C2—C3	178.82 (16)	C2—C1—C11—O1	109.0 (2)
O2—C2—C3—C4	−177.90 (18)	C10—C1—C11—O1	−72.7 (2)
C1—C2—C3—C4	1.0 (3)	C2—C1—C11—C12	−71.7 (2)
C2—C3—C4—C5	−1.1 (3)	C10—C1—C11—C12	106.57 (18)
C3—C4—C5—C10	−0.4 (3)	O1—C11—C12—C13	161.18 (18)
C3—C4—C5—C6	−177.56 (19)	C1—C11—C12—C13	−18.1 (2)
C4—C5—C6—C7	175.11 (19)	O1—C11—C12—C17	−17.2 (3)
C10—C5—C6—C7	−2.1 (3)	C1—C11—C12—C17	163.60 (16)
C5—C6—C7—C8	−0.4 (3)	C17—C12—C13—C14	−0.3 (3)
C20—O3—C8—C9	−2.8 (3)	C11—C12—C13—C14	−178.72 (16)
C20—O3—C8—C7	177.68 (16)	C12—C13—C14—C15	−0.8 (3)
C6—C7—C8—O3	−178.08 (17)	C13—C14—C15—C16	1.0 (3)
C6—C7—C8—C9	2.3 (3)	C13—C14—C15—Cl1	−179.07 (14)
O3—C8—C9—C10	178.81 (16)	C14—C15—C16—C17	0.0 (3)
C7—C8—C9—C10	−1.7 (3)	Cl1—C15—C16—C17	−179.94 (14)
C8—C9—C10—C5	−0.9 (3)	C15—C16—C17—C12	−1.2 (3)
C8—C9—C10—C1	−177.22 (16)	C13—C12—C17—C16	1.4 (3)
C4—C5—C10—C9	−174.63 (16)	C11—C12—C17—C16	179.72 (16)
C6—C5—C10—C9	2.7 (2)	C2—O2—C18—C19	−162.1 (2)
C4—C5—C10—C1	1.9 (2)		

### Hydrogen-bond geometry (Å, °)

**Table:**

*D*—H···*A*	*D*—H	H···*A*	*D*···*A*	*D*—H···*A*
C20—H20B···Cg1^i^	0.98	3.02	3.821 (3)	140
C20—H20C···Cg1^ii^	0.98	3.01	3.477 (3)	110
C13—H13···O3^iii^	0.95	2.44	3.213 (2)	138

Symmetry codes: (i) *x*, −*y*+1/2, *z*+1/2; (ii) *x*+1, −*y*+1/2, *z*+1/2; (iii) *x*, −*y*+1/2, *z*−1/2.

## References

[bb1] Burla, M. C., Caliandro, R., Camalli, M., Carrozzini, B., Cascarano, G. L., De Caro, L., Giacovazzo, C., Polidori, G. & Spagna, R. (2005). *J. Appl. Cryst.* **38**, 381–388.

[bb2] Burnett, M. N. & Johnson, C. K. (1996). *ORTEPIII* Report ORNL-6895. Oak Ridge National Laboratory, Tennessee, USA.

[bb3] Higashi, T. (1999). *NUMABS* Rigaku Corporation, Tokyo, Japan.

[bb4] Mitsui, R., Nakaema, K., Noguchi, K., Okamoto, A. & Yonezawa, N. (2008). *Acta Cryst.* E**64**, o1278. 10.1107/S1600536808017297 PMC2961834 21202910

[bb5] Mitsui, R., Nakaema, K., Noguchi, K. & Yonezawa, N. (2008). *Acta Cryst.* E**64**, o2497. 10.1107/S1600536808039603 PMC2959982 21581458

[bb6] Rigaku (1998). *PROCESS-AUTO* Rigaku Corporation, Tokyo, Japan.

[bb7] Rigaku/MSC (2004). *CrystalStructure* Rigaku/MSC, The Woodlands, Texas, USA.

[bb8] Sheldrick, G. M. (2008). *Acta Cryst.* A**64**, 112–122. 10.1107/S0108767307043930 18156677

